# Complex Presentation of Goldenhar Syndrome in a Preterm Neonate: A Case Report

**DOI:** 10.7759/cureus.63624

**Published:** 2024-07-01

**Authors:** Vasu Saini, Himani Sharma, Anjani Mahesh Kumar Cherukuri, Chaitanya Kumar Javvaji, Bhumika Bheemavarapu

**Affiliations:** 1 Pediatrics, Shri Guru Ram Rai Institute of Medical and Health Sciences, Dehradun, IND; 2 Otolaryngology - Head and Neck Surgery, Noida International Institute of Medical Sciences, Greater Noida, IND; 3 Pediatrics, Guntur Medical College, Guntur, IND; 4 Pediatrics, Jawaharlal Nehru Medical College, Datta Meghe Institute of Higher Education and Research, Wardha, IND; 5 Pediatrics, Jawaharlal Institute of Postgraduate Medical Education and Research, Pondicherry, IND

**Keywords:** congenital anomalies, ventricular septal defect (vsd), anotia, duodenal atresia, goldenhar syndrome

## Abstract

Goldenhar syndrome, also known as oculo-auriculo-vertebral syndrome, is a rare congenital disorder characterized by craniofacial anomalies, ear malformations, and ocular abnormalities. It is also associated with multiple system involvement, including the central nervous system, renal, cardiovascular, and gastrointestinal systems. This case report presents a detailed description of a preterm female neonate diagnosed with Goldenhar syndrome. Many of the classical features, along with ventricular septal defect (VSD), were present in our patient. She was complicated by prematurity and a urinary tract infection and was later diagnosed with a VSD at the age of three months. The multidisciplinary examination and management involving pediatricians, pediatric surgeons, ophthalmologists, and otorhinolaryngologists led to comprehensive care for the patient. This case emphasizes the importance of early diagnosis and management for optimal patient outcomes.

## Introduction

Goldenhar syndrome, first described by French ophthalmologist Maurice Goldenhar in the early 1950s, is a rare congenital disorder primarily involving craniofacial anomalies [1]. Goldenhar reported a triad of accessory tragi, ocular dermoid, and mandibular hypoplasia. Gorlin et al. reported the association of vertebral anomalies with Goldenhar syndrome, eventually leading to the name oculo-auriculo-vertebral syndrome [2]. Goldenhar syndrome is also associated with multiple system involvement. The definitive cause of the disease has not been identified yet. The variability and severity of symptoms make diagnosis challenging, often requiring a multidisciplinary collaborative approach. This case report aims to highlight the clinical features, diagnostic modalities, and management strategies employed in the care of a patient diagnosed with Goldenhar syndrome who presented with duodenal atresia.

## Case presentation

**Figure 1:**
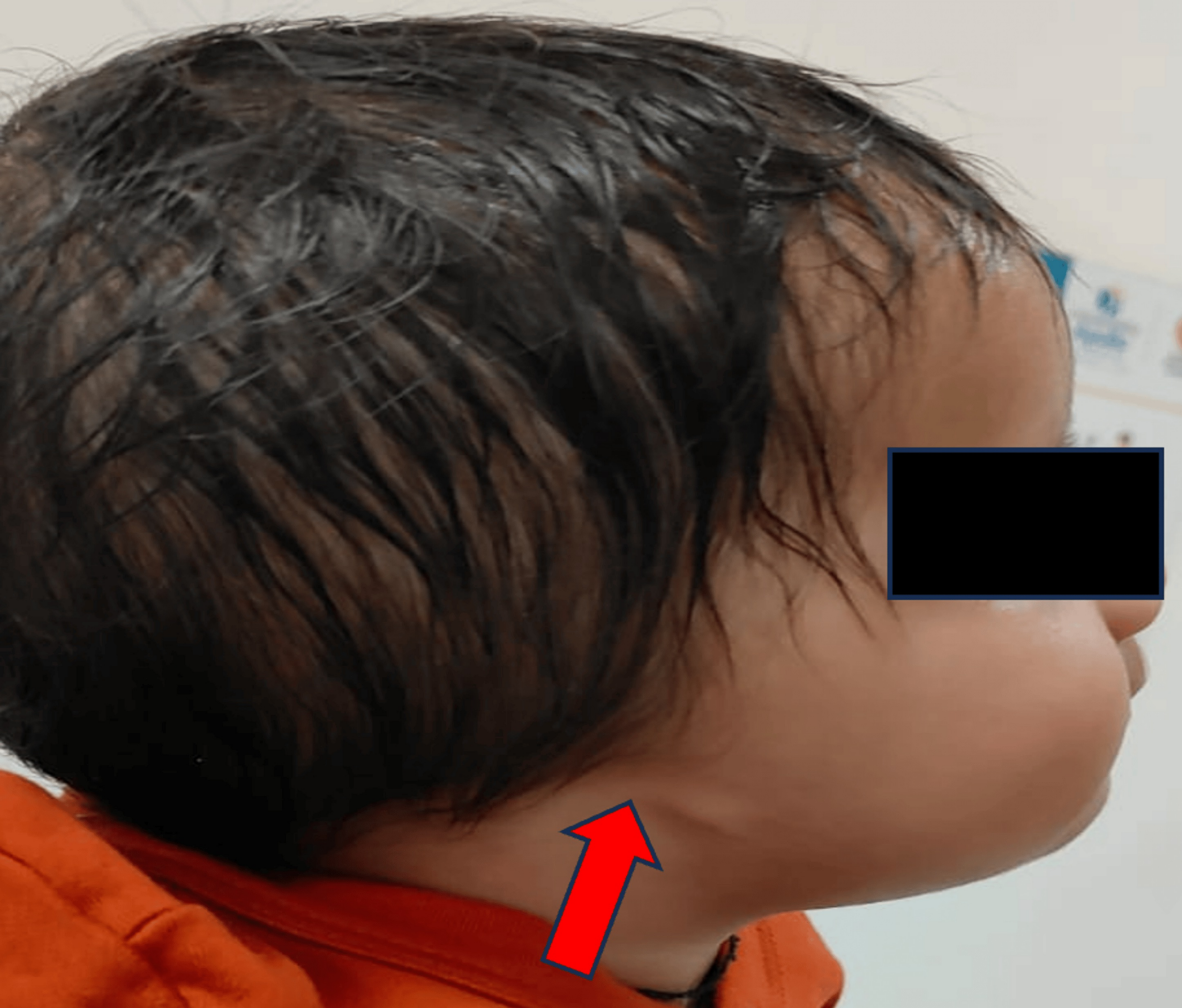
Clinical image showing right anotia (red arrow)

**Figure 2:**
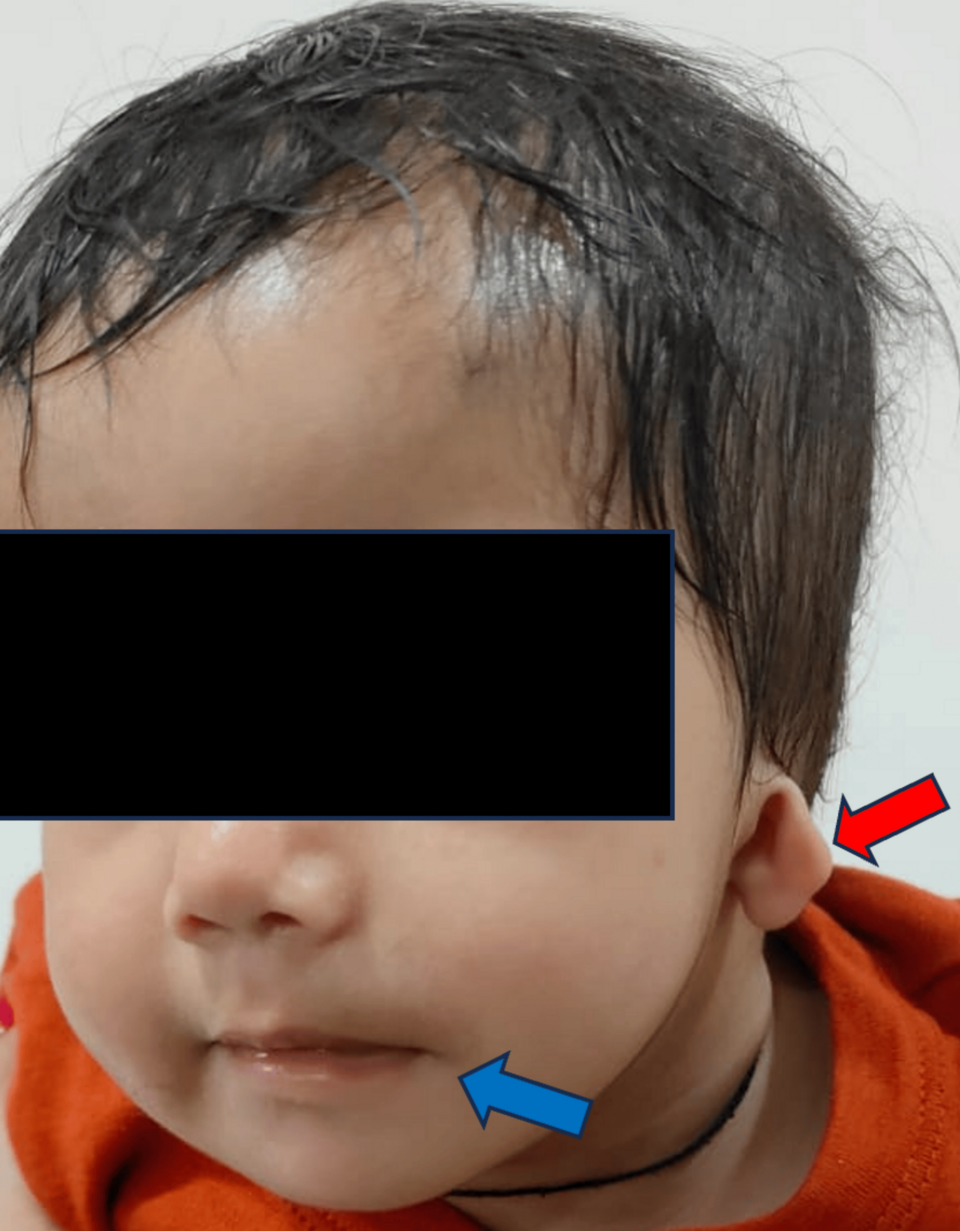
Clinical image showing a malformed left ear (red arrow) and a slight deviation of the angle of the mouth towards the left (blue arrow)

An eight-month-old female born out of non-consanguineous pregnancy to a 23-year-old primigravida. The pregnancy was notable for preterm labor, culminating in a premature lower segment cesarean section at 35 weeks and two days. No medications, known teratogenic exposures, or illnesses were reported during the pregnancy. At birth, the infant's heart rate was initially 80 beats per minute but improved following bag and mask ventilation. There was no family history of congenital disabilities, and both parents exhibited normal facial development. The neonate presented with multiple congenital anomalies, including a high-arched palate, right anotia, a malformed left ear, and a slight deviation of the mouth angle towards the left side (Figures 1-2).

**Figure 3:**
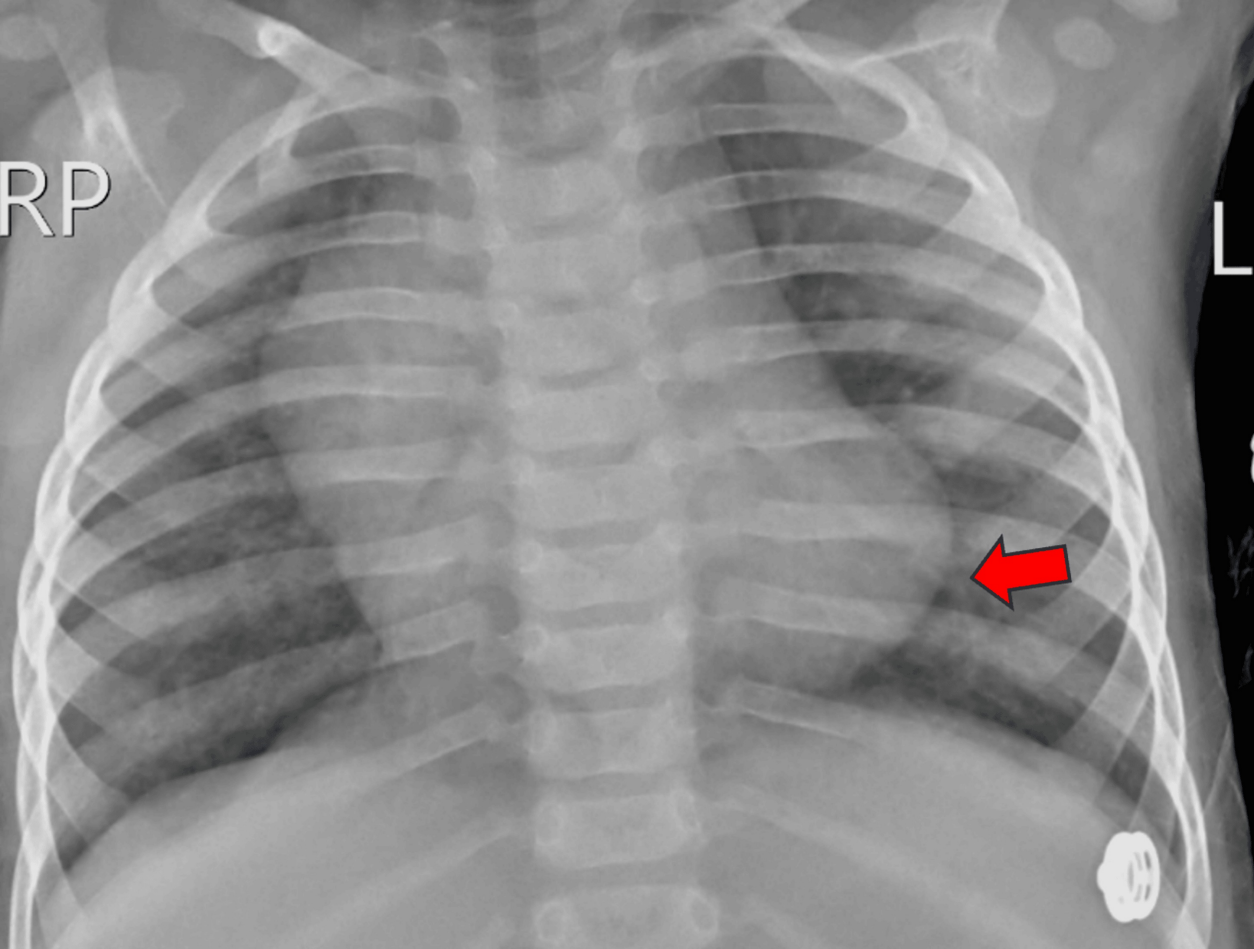
Chest X-ray showing cardiomegaly (red arrow)

A cardiovascular examination revealed a holosystolic murmur at the lower left sternal border. A chest X-ray demonstrated cardiomegaly (Figure 3).

**Figure 4:**
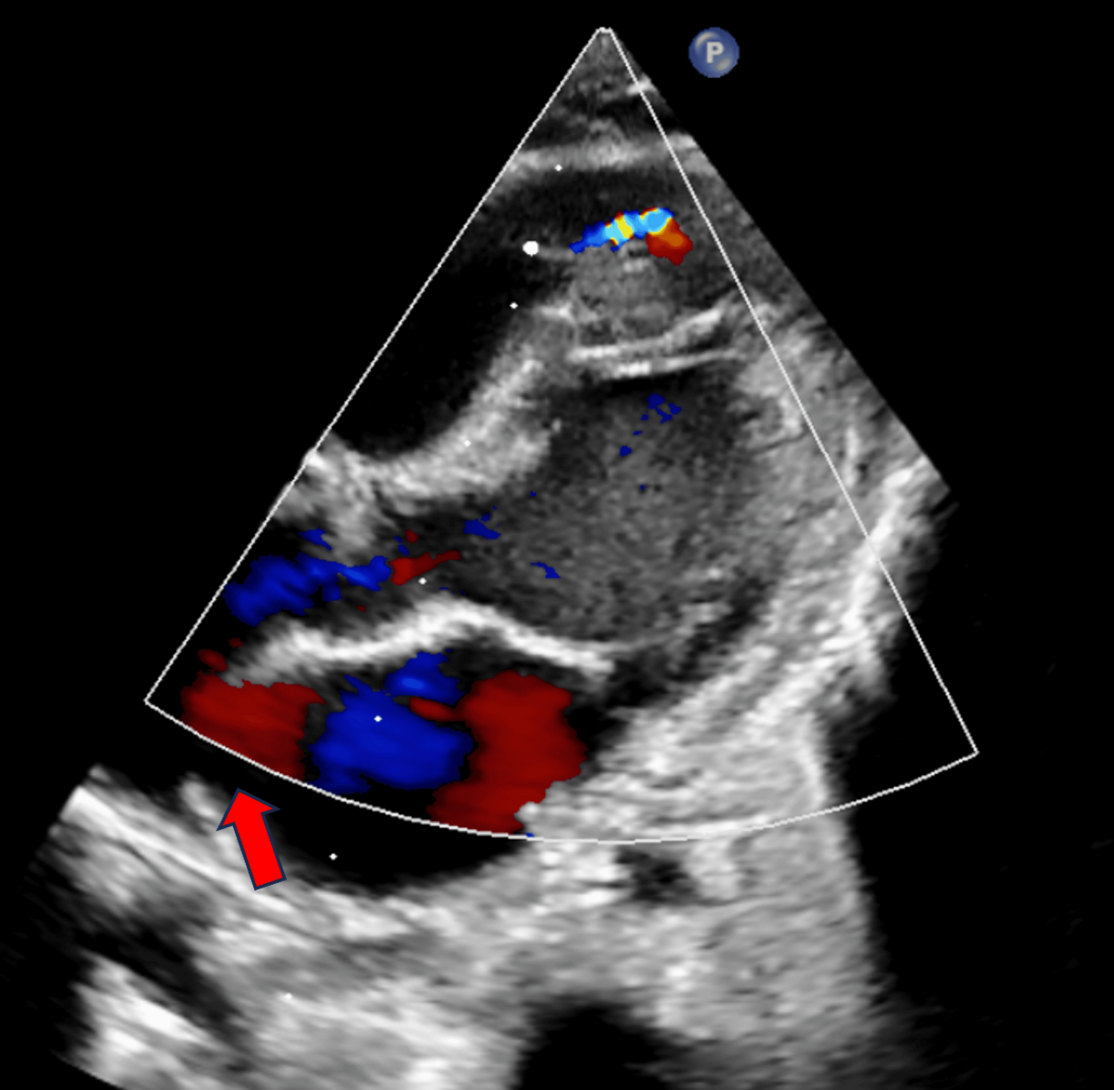
2D echocardiography indicating a ventricular septal defect (red arrow)

A 2D echocardiogram indicated a ventricular septal defect (VSD) with a gradient of 67 mmHg, a left-to-right shunt, and mild left atrial dilatation (Figure 4).

Ultrasonography of the kidneys, ureters, and bladder showed bilateral dilated pelvicalyceal systems with mild renal parenchymal thinning. A urine culture identified *Klebsiella pneumoniae* growth. Despite this, the sepsis profile was normal. On the 15^th^ day of admission, otoacoustic emission testing revealed that the left ear had abnormal function of outer hair cells and the right ear's external auditory canal was absent. The retinopathy of prematurity screening was normal. A repeat urine culture returned normal results. Hammersmith's infant neurological examination score was 28.

The patient received intravenous antibiotics for the urinary tract infection and intravenous fluids and orogastric feeds to maintain hydration and nutrition. The patient remained hemodynamically stable and was subsequently discharged. Based on the clinical findings and diagnostic investigations, Goldenhar syndrome was diagnosed. The diagnostic criteria met included facial asymmetry, ear malformations, ocular abnormalities, and other significant organ anomalies. Regular follow-up appointments were scheduled to monitor progress, ensure optimal development, and address any emerging issues promptly.

## Discussion

Goldenhar syndrome is a rare congenital disease with an incidence of one in 3,500 to one in 25,000 babies with a 3:2 male preponderance [3,4]. No definitive etiopathogenesis of the disease has been identified, but it is proposed to be multifactorial with most cases being sporadic [5]. Familial inheritance, although rare, has been observed [6]. The proposed causative factors contribute to the pathogenesis by disrupting neural crest cell migration and abnormal vascular supply to branchial arches, leading to developmental defects in the derivatives of the first and second branchial arches [7-11].

Although definitive causes of the disease have not been identified, a few are genetic like deletion of 5p, duplication of 14q23.1, and abnormalities of chromosomes 18 and 22 [10-12]. Prenatal associations have been observed, such as maternal diabetes, maternal use of vasoactive drugs, maternal second-trimester bleeding, and maternal use of assisted reproductive technology and retinoids [4,11,13]. It has also been associated with antibodies against cytomegalovirus, herpes simplex virus, and rubella virus [14]. Individuals with Goldenhar syndrome typically present during childhood with facial asymmetry, ocular defects, hearing problems, feeding difficulties, and scoliosis [15].

They may develop intellectual disability and developmental and behavioral problems [10]. The disease can also affect multiple systems, including the cardiac, renal, gastrointestinal, and central nervous systems (CNS) [16]. Our case had right anotia, malformation of the left external ear, minimally noticeable facial asymmetry, VSD, and bilateral pelvicalceal dilation with mild renal parenchymal thinning. The condition of the neonate in our case was far more critical due to prematurity and urinary tract infection. The disease diagnosis is primarily based on clinical features and imaging. The milder forms of the disease usually go undetected during the early stages of life [17]. The presence of a life-threatening complication, such as duodenal atresia in our case, prompts a thorough examination, yielding an early diagnosis of the disease.

Multiple investigations, such as ultrasound imaging and genetic analysis, are helpful in the prenatal diagnosis of Goldenhar syndrome. In a study by Barisic et al., it was observed that ultrasound imaging helped in the prenatal diagnosis of 19% of cases with Goldenhar syndrome, where prenatal ultrasound imaging showed asymmetrical craniofacial defects, CNS, congenital heart, genitourinary, and spine anomalies [18]. Examination of the neonate in our case did not show any vertebral dysplasia or scoliosis. Regular follow-ups are recommended for our case to monitor for the development of any vertebral anomalies. Early diagnosis of such could prevent the development of disability in the child by prompting timely intervention.

Literature suggests that the child can develop hearing loss, and early intervention addressing the hearing impairment may aid in cognitive development [19]. The child may require a few corrective surgeries to her face depending on the impairment due to the facial defects. Timed follow-ups with a multidisciplinary team involving pediatricians, ophthalmologists, otorhinologists, speech and behavioral therapists, orthodontists, and maxillofacial surgeons will be an efficient method to ensure well-rounded development of the child.

## Conclusions

In conclusion, this case highlights the complex presentation of Goldenhar syndrome in a preterm neonate, characterized by multiple congenital anomalies including facial asymmetry, ear malformations, and cardiovascular defects. Early diagnosis and prompt management, including the treatment of associated infections and supportive care, played a crucial role in stabilizing the patient's condition. The patient's significant improvement and overall well-being underscore the importance of regular follow-up to monitor development and address emerging issues. This case underscores the need for a multidisciplinary approach to managing Goldenhar syndrome to optimize outcomes and ensure comprehensive care for affected individuals.
